# The diagnostic accuracy of isothermal nucleic acid point-of-care tests for human coronaviruses: A systematic review and meta-analysis

**DOI:** 10.1038/s41598-020-79237-7

**Published:** 2020-12-18

**Authors:** Pakpoom Subsoontorn, Manupat Lohitnavy, Chuenjid Kongkaew

**Affiliations:** 1 Department of Biochemistry, Faculty of Medical Science, Naresuan University, Phitsanulok, 65000 Thailand; 2 Department of Pharmacy Practice, Faculty of Pharmaceutical Sciences, Naresuan University, Phitsanulok, Thailand; 3 Center of Excellence for Environmental Health and Toxicology, Faculty of Pharmaceutical Sciences, Naresuan University, Phitsanulok, 65000 Thailand; 4 Research Centre for Safety and Quality in Health, Faculty of Pharmaceutical Sciences, Naresuan University, Phitsanulok, 65000 Thailand; 5 Research Department of Practice and Policy, UCL School of Pharmacy, 29-39 Brunswick Square, London, WC1N 1AX UK

**Keywords:** Biochemistry, Biotechnology, Microbiology, Molecular biology, Diseases, Molecular medicine

## Abstract

Many recent studies reported coronavirus point-of-care tests (POCTs) based on isothermal amplification. However, the performances of these tests have not been systematically evaluated. Cochrane Handbook for Systematic Reviews of Diagnostic Test Accuracy was used as a guideline for conducting this systematic review. We searched peer-reviewed and preprint articles in PubMed, BioRxiv and MedRxiv up to 28 September 2020 to identify studies that provide data to calculate sensitivity, specificity and diagnostic odds ratio (DOR). Quality Assessment of Diagnostic Accuracy Studies 2 (QUADAS-2) was applied for assessing quality of included studies and Preferred Reporting Items for a Systematic Review and Meta-analysis of Diagnostic Test Accuracy Studies (PRISMA-DTA) was followed for reporting. We included 81 studies from 65 research articles on POCTs of SARS, MERS and COVID-19. Most studies had high risk of patient selection and index test bias but low risk in other domains. Diagnostic specificities were high (> 0.95) for included studies while sensitivities varied depending on type of assays and sample used. Most studies (n = 51) used reverse transcription loop-mediated isothermal amplification (RT-LAMP) to diagnose coronaviruses. RT-LAMP of RNA purified from COVID-19 patient samples had pooled sensitivity at 0.94 (95% CI: 0.90–0.96). RT-LAMP of crude samples had substantially lower sensitivity at 0.78 (95% CI: 0.65–0.87). Abbott ID Now performance was similar to RT-LAMP of crude samples. Diagnostic performances by CRISPR and RT-LAMP on purified RNA were similar. Other diagnostic platforms including RT- recombinase assisted amplification (RT-RAA) and SAMBA-II also offered high sensitivity (> 0.95). Future studies should focus on the use of un-bias patient cohorts, double-blinded index test and detection assays that do not require RNA extraction.

## Introduction

Coronavirus epidemics have caused serious damage to public health and the global economy. Severe acute respiratory syndrome coronavirus (SARS-CoV) and Middle East respiratory syndrome coronavirus (MERS-CoV) infected over ten thousand people and killed over a thousand people worldwide^1^. A novel coronavirus (SARS-CoV-2) that causes coronavirus disease 2019 (COVID-19) infected over 60 million people and so far killed over 1,400,000 people (as of Nov 26th, 2020). The global GDP is predicted to shrink by almost one percent^2^. Rapid and low-cost diagnostic screening of a population at risk is critical for controlling sources of infection. Such diagnostic capability also helps policy makers decide when and to what extent to ease restrictions and restore the economy^3^.

Reverse transcription quantitative polymerase chain reaction (RT-qPCR) has been the gold standard for RNA virus detection^4,5^. Nonetheless, RT-qPCR requires up to 4 h sample-to-result time and needs a bulky expensive thermal cycler with fluorimetry. To fulfil the demand for rapid diagnoses during disease outbreaks, point-of-care tests (POCTs) are needed that are cheaper, faster and deployable in the field.

Nucleic acid detections based on isothermal amplification obviate the need for a thermal cycler thereby simplifying and speeding up the diagnosis process. For instance, loop-mediated isothermal amplification (LAMP) relies on strand displacing DNA polymerase and primers to amplify specific DNA sequences of pathogens^6^. Reverse transcription LAMP (RT-LAMP) has been applied for the detection of various RNA viruses including Ebola, Zika, West Nile, Influenza and Yellow fever viruses^7–11^. Rolling circle amplification (RCA) utilizes highly processive strand displacement DNA polymerase and circularizable oligonucleotide probes for detecting single strand DNA or RNA^12^. Reverse transcription insulated isothermal PCR (RT-iiPCR) relies on a temperature gradient to drive denaturation/annealing/extension cycle similar to conventional PCR but in the absence of a thermal cycler^13^. Reverse transcription recombinase polymerase amplification (RT-RPA) or reverse transcription recombinase aided amplification (RT-RAA) uses recombinase, single strand binding protein, DNA polymerase and reverse transcriptase to amplify the RNA target^14^. Simple amplification based assay (SAMBA) uses DNA dependent RNA polymerase and RNA dependent DNA polymerase to alternately transcribe and reverse transcribe RNA target^15^. CRISPR diagnosis combines isothermal amplification techniques (such as RT-LAMP and RT-RPA) with specific DNA or RNA targeting ability of crRNA and Cas12 or Cas13 enzymes^16^. The outputs of these detection techniques can be coupled with fluorescent or colorimetric reporters as well as lateral flow strip platforms to facilitate readout processes.

While many studies presented nucleic acid POCTs for human coronaviruses, it is important to systematically evaluate and draw conclusions about the performance of POCTs and quality of these studies. These could guide clinical practice and highlight opportunities for next generation POCTs. Here, we aim to determine the accuracy of nucleic acid point-of-care diagnosis for human coronaviruses, particularly, SARS-CoV, MERS-CoV and SARS-CoV-2, using systematic review and meta-analysis techniques.

## Methods

This study followed the guidelines in the ‘Cochrane Handbook for Systematic Reviews of Diagnostic Test Accuracy’^17^.

### Eligibility criteria

#### Inclusion criteria

This systematic review and meta-analysis included (1) both peer-reviewed and preprint original articles on nucleic acid based POCTs; (2) the test must be isothermal, i.e., thermal cycling is not required during the test; (3) full text is available (in any language); and (4) provide enough information to determine the number of true positive, false positive, false negative and true negative on POCTs (performed on clinical samples) relative to a standard reference test.

#### Exclusion criteria

We excluded (1) studies investigating antibody test, direct antigen tests or non-isothermal nucleic acid test, (2) studies that did not use clinical samples.

### Search strategy

Peer-reviewed articles were searched on PubMed from its inception up to 28 September 2020 with the following search terms: (coronavirus OR COVID-19 OR severe acute respiratory syndrome OR middle east respiratory syndrome) AND (rapid diagnosis OR isothermal amplification). Preprint articles were searched on BioRxiv and MedRxiv from 1 January 2020 to 28 September 2020 using a search term ‘isothermal amplification’. The titles, abstracts and duplicates were screened and the full text of relevant articles were reviewed by PS and cross-checked by CK. We registered our systematic review and meta-analysis on PROSPERO on April 21, 2020; registration number yet to be updated.

### Quality assessment

The quality of each study was assessed with the Quality Assessment of Diagnostic Accuracy Studies 2 (QUADAS-2)^18^. QUADAS-2 consists of four key domains: (1) patient selection; (2) index test; (3) reference standard; (4) flow and timing. Each is assessed in terms of risk of bias and the first three in terms of concerns regarding applicability. These domains were assessed by using 18 signalling questions with ‘yes’, ‘no’ and ‘unclear’ answers. Specific criteria for what qualified as ‘yes’, ‘no’ and ‘unclear’ are shown (Table S1). Then, the answers were used to judge whether the risk of bias and the concern for the applicability of the research is low, high or unclear. Specific criteria for each domain i.e., what qualified as high or low risk of bias are shown (Table S2). Two reviewers (PS and CK) independently judged the quality of each study. Disagreements were resolved by consensus with additional input from ML.

### Data extraction

Data were extracted by one reviewer (PS) and where the results were unclear, the two other reviewers (CK and ML) were consulted. The parameters extracted included: citation information, types of coronaviruses, methodology, and the diagnostic accuracy of results (Table S3).

Handling articles during data extraction:

1. For studies performing on different sets of samples (e.g. different patient groups from different hospitals) using the same diagnosis assays and parameter settings, we included all studies separately.
2. For studies performing on the same sets of samples using different diagnosis assays (e.g. CRISPR diagnosis vs RT-LAMP) or different variants of the same assays (e.g. using crude samples vs purified RNA or using fluorescent readout vs lateral flow strip test), we included all studies separately. Notably, redundant samples were excluded in subgroup analysis.
3. For studies performing on the same set of samples using the same diagnosis assay but different parameter settings (e.g. using different incubation times and temperatures), we included only the study that reported the highest sensitivity and specificity.

### Statistical data analysis and reporting

Forest plots were generated and pooled sample statistics were calculated using R packages ‘mada’^19^ in R program (version 4.0.0)^20^. To avoid statistical artefacts from cells containing zero values in a 2 × 2 table (for example when false positive or false negative are zero), continuity corrections = 0.5 were added to the observed frequencies when calculating diagnostic odds ratio (DOR)^19^. Since the sensitivity and specificity of a diagnostic test depend on each other, bivariate approaches to the meta-analysis of diagnostic accuracy was recommended for estimating sensitivity, specificity and DOR in the ‘mada’ package.

Since Deeks’ test is recommended for diagnostic test accuracy (DTA) meta-analyses^21^ formal testing for publication bias was undertaken by a regression of diagnostic log odds ratio against 1/sqrt (effective sample size), weighted by effective sample size, with P < 0.10 for the slope coefficient indicating significant asymmetry^22^.

The Preferred Reporting Items for a Systematic Review and Meta-analysis of Diagnostic Test Accuracy Studies’ (PRISMA-DTA) was used for reporting^23^.

## Results

### Search results

**Figure 1:**
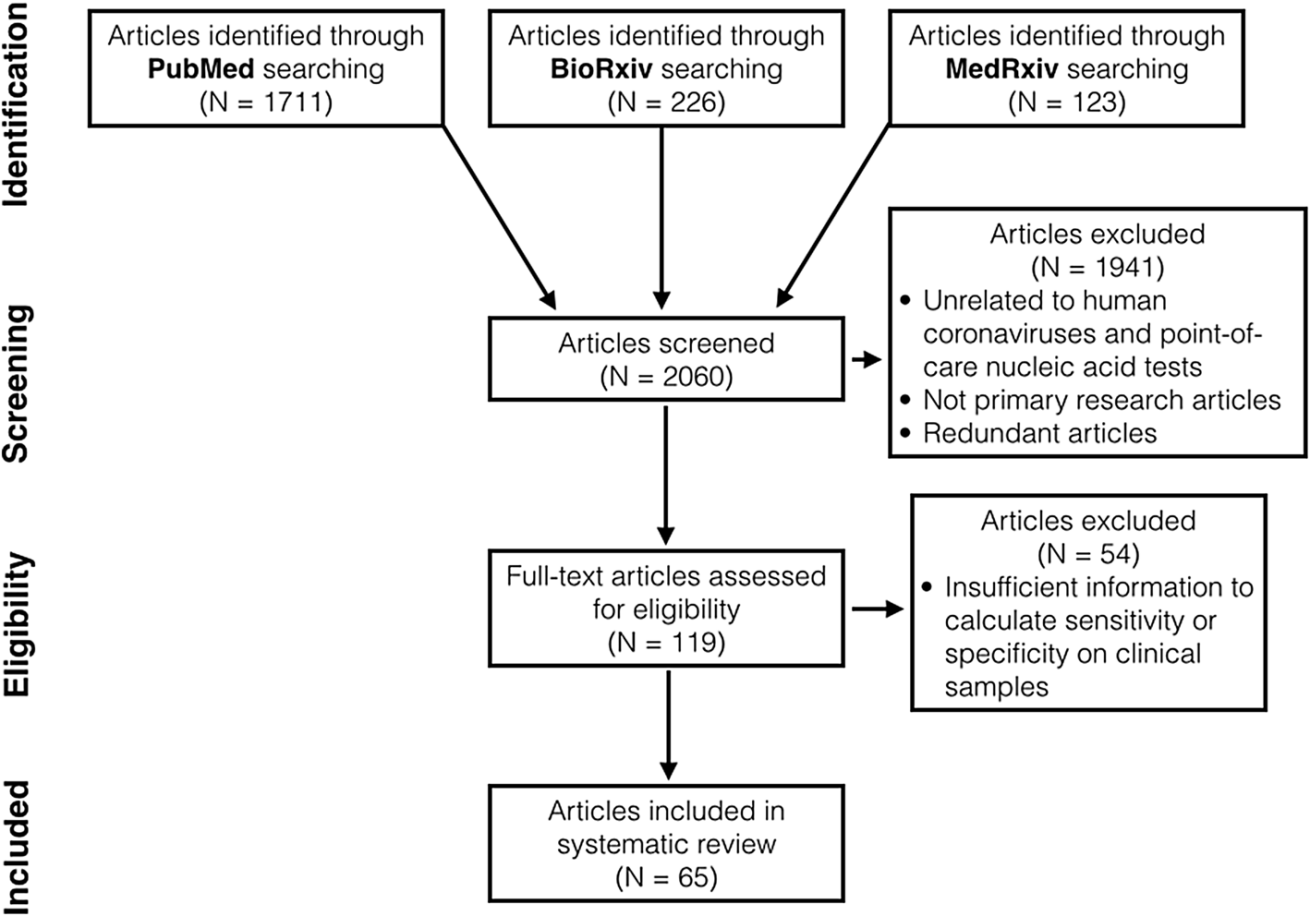
The preferred reporting items for a systematic review and meta-analysis (PRISMA) flow diagram.

We identified 2060 articles in total through database searching (Fig. 1). After title and abstract screening, we excluded 1941 articles that were not primary research articles, had no full text available or were unrelated to nucleic acid POCTs for human coronaviruses. 62 non-English articles were found but only two met above eligibility criteria. These two articles were later excluded as the authors did not use clinical samples. After reviewing full text, we found 65 articles with sufficient information to calculate sensitivity, specificity and diagnostic odds ratio (DOR) on clinical samples^24–88^ (Table S3). Of 65 articles, 13 reported more than one study conducted on different sample groups or using different diagnostic procedures. In total, we included 81 studies in our systematic review.

### Characteristics of the included studies

Most studies used clinical samples from USA (n = 20 out of 81 studies), followed by China (n = 17) and UK (n = 10). Most studies (n = 76) were COVID-19 diagnostic studies published or uploaded to preprint databases in 2020. Most studies (n = 51) use RT-LAMP as nucleic acid POCTs, followed by CRISPR diagnosis (n = 12), RT-RPA/RAA (n = 7), Abbot ID Now (n = 5), and SAMBA II (n = 2). The rest were iAMP, RT-iiPCR, RT-MDCA, and RCA (n = 1 each). Over a third (n = 28) of all studies attempted to diagnose coronaviruses in crude patient samples, i.e., nasopharyngeal swabs, sputum, saliva, etc. The others (n = 53) used purified RNA from patient samples for viral diagnosis.

### Quality of articles

**Figure 2:**
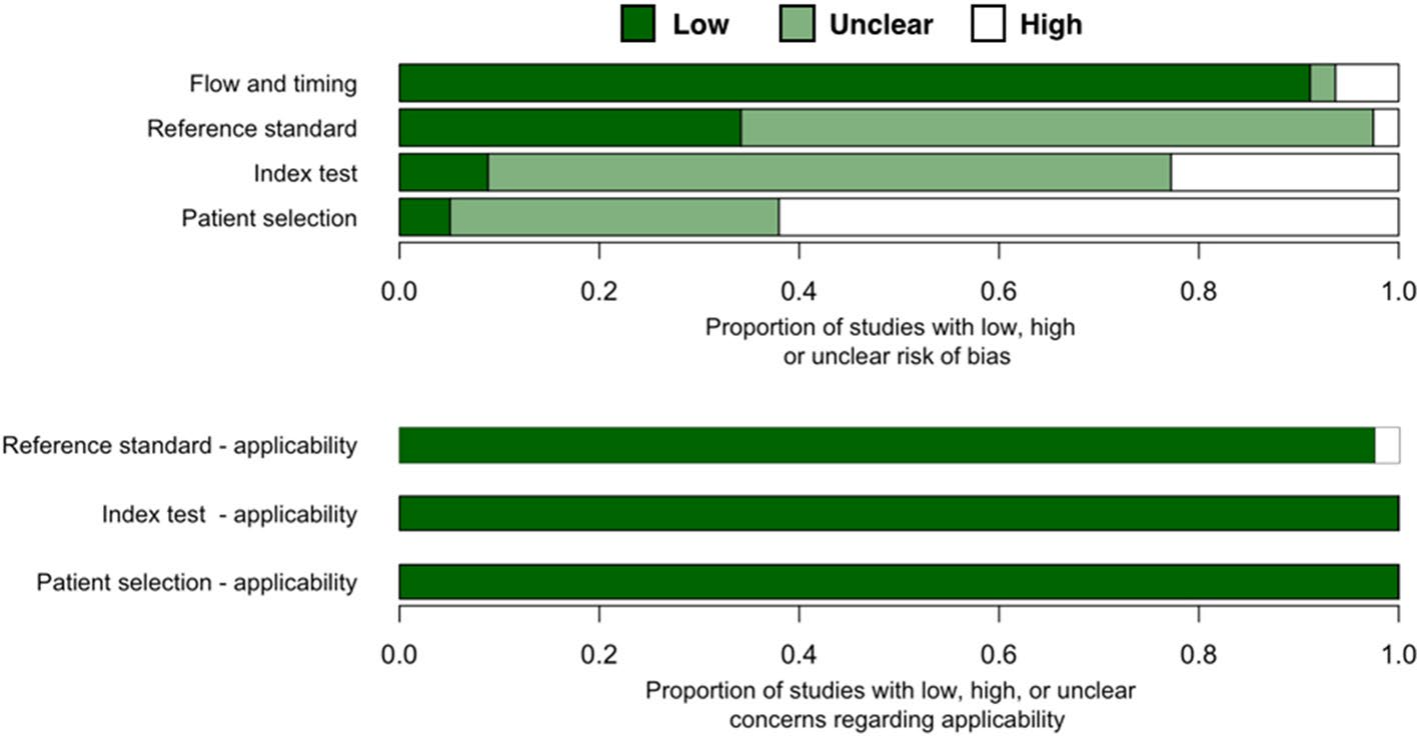
Quality assessment of diagnostic accuracy studies 2 (QUADAS-2) finding per domain for 81 studies included in this systematic review.

Almost two thirds of all studies (n = 50 out of 81 studies) have high risk of patient selection bias due to non-random patient selection and case–control study design (Fig. 2, Table S2). These studies specifically recruited clinical samples known to be uninfected or infected with coronavirus. Over a third of all studies have unclear risk of patient selection bias because these studies were not case–control but provided insufficient detail about patient inclusion/exclusion criteria. Only four studies^38,49,65^ has low risk of patient selection bias.

Over one fifth of all studies (n = 18 out of 81 studies) have high risk of index test bias because these tests used qualitative fluorescent or colorimetric readout without defined detection thresholds. Only seven studies had low risk of index test bias as these studies had quantitative detection readouts with reported thresholds. These studies also explicitly declared that their index and reference tests were done simultaneously/in parallel to each other or that testing was blinded from each other. For other studies (n = 56), it was either unclear whether index test results were interpreted with knowledge of reference test results or qualitative readout was used for interpreting the results. Thus, index test bias of these studies are unclear.

Only two studies have high risk of reference standard bias as they used RT-PCR (not quantitative, readout result in agarose gel electrophoresis)^47^ or immunofluorescent assay (IFA)^69^ as a reference standard test. For the rest of included studies, almost two thirds (n = 52) have unclear risk of reference standard bias because these studies did not provide enough information about whether reference standard results were interpreted without knowledge of the results of the index test.

Most studies have a low risk of flow and timing bias with the following exceptions. One study provided no information on whether the samples for a reference test (IFA) and the index test (RT-LAMP) were taken at the same time^69^. Another study might have excluded some samples from the workflow^87^. These two studies were marked as having unknown risk of flow and timing bias. Three studies were designated as having high risk because they used different standard references on different samples^80^, used different samples test flow on different sample groups^65^, and excluded some samples from the analysis^76^ (Table S2).

Our review question did not focus on any particular patient demographics. None of the included studies attempted to exclude patients based on demographics and thus had no ‘concern of patient selection applicability’(Fig. 2, Table S2). Index isothermal tests of all studies have generally been used for POCTs and thus have low concern of index test applicability. Reference standard tests of nearly all studies are RT-qPCR, the current gold standard for RNA virus detection. Thus, we graded these studies as having low concern of standard test applicability. Two studies that used (non-quantitative) RT-PCR^47^ and IFA^69^, were marked as having high concern of standard test applicability.

### Sensitivity, specificity and diagnostic odd ratio (DOR) of nucleic acid POCTs

**Figure 3:**
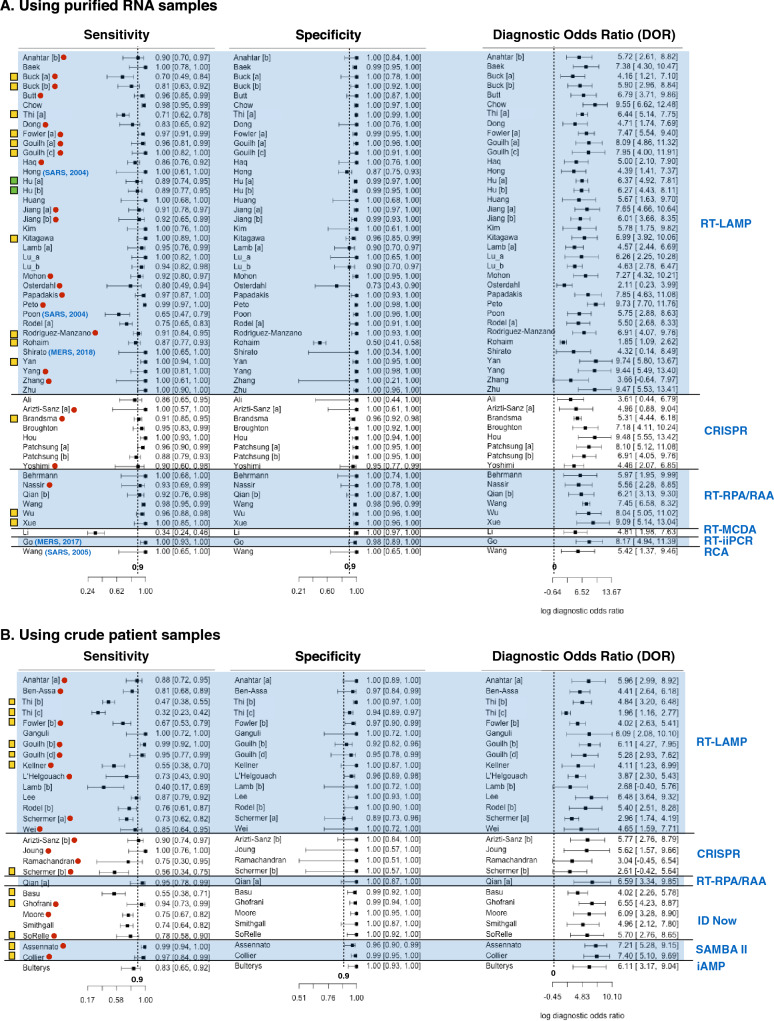
The forest plot of sensitivity, specificity and diagnostic odds ratio (DOR) of human coronavirus nucleic acid point-of-care tests (POCTs) on purified RNA samples **(A)** and on crude patient samples **(B).** Rows shows first author name, and performance (sensitivity, specificity and DOR) of each study. Different studies from the same research articles are labelled with different letter [a], [b], [c], etc. Blue parentheses after first author names indicates the types of coronaviruses diagnosed and publication years of the studies. All rows without blue parentheses show studies on COVID-19 diagnosis published in 2020. Red dots indicate those studies that were only available as pre-prints (not peer-reviewed). The far right blue texts indicate the types of diagnostic assays used in the studies. The far left yellow boxes prefacing the author names denote the studies having no QUADAS-2 domain with high risk bias or high concern of applicability but have unclear bias or concerns in some QUADAS-2 domain. Green boxes denote that the study has low risk of bias and low concern of applicability in all QUADAS-2 domains. All rows without yellow or green box show studies with high risk of bias or high concern of applicability in at least one QUADAS-2 domain.

Nearly all studies (n = 77 out of 81) reported at least 90% diagnosis specificity while less than two third (n = 48 out of 81) reported 90% sensitivity or above (Fig. 3). Less than a third (n = 14 out of 53) of studies that used purified RNA for diagnosis reported below 90% sensitivity (Fig. 3A). In contrast, over two thirds (n = 19 out of 28) of studies that used crude patient samples for diagnosis reported sensitivities less than 90% (Fig. 3B). Thus, for most studies, diagnostic specificity is of less concern than sensitivity. Moreover, diagnostic sensitivity of purified RNA is generally higher than those of crude patient samples. All studies reported DOR above one.

Among studies that used RT-LAMP on purified RNA samples, the study by Rohaim et al. (2020) is clearly an outlier (Fig. 3A)^74^. This study used artificial intelligence to interpret the RT-LAMP colorimetric readout. While this approach can reduce assay time and eliminate subjectivity of result interpretation, the false positive rate was high (approximately 50% when using RT-qPCR as a reference test). Osterdahl et al. (2020) is the only study whose both sensitivity and specificity were 80% or below^65^. This study had high risk of flow and timing bias because some clinical samples were taken on different days for index test and standard reference test. Poon et al. (2004) reported the lowest sensitivity (at 65%) among all studies using RT-LAMP on purified RNA samples^69^. This study has a high risk of reference test bias and high concern of reference test applicability because immunofluorescent assay (IFA) was used for reference test instead of RT-qPCR. Since the antibody may persist much longer in patients than the virus. As a result, some samples might give a positive result to the antibody test but provide a negative result to the LAMP test. Buck et al. (2020), Thi et al. (2020) and Rodel et al. (2020) are also studies with sensitivity below 80%^34,39,72^. These studies also reported quantity of viral RNA (as Ct value of RT-qPCR) in purified RNA sample. The authors showed that that samples with low viral RNA (i.e. high Ct value above 30) accounted for a significant portion of coronavirus positive samples used in the studies. Since these samples were more difficult to detect (i.e. more likely to get false negative), this could explain apparently low sensitivities reported by these three studies.

For diagnosis of purified RNA samples using non RT-LAMP assays, all studies using RT-RPA/RAA, CRISPR diagnosis, RT-iiPCR, and RCA as index tests reported sensitivity and specificity at close to 90% or above (Fig. 3A). The Li et al. (2020)^59^ study is clearly an outlier. The study introduced a new diagnosis assay called reverse transcription multiple cross displacement amplification (RT-MCDA). The authors claimed that this new assay was more sensitive than RT-qPCR. Nonetheless, the result showed that RT-MCDA could only detect viral RNA in 33.8% of COVID-19 confirmed patient samples. Such low sensitivity could result from the performance of RT-MDCA itself or the fact that viral RNA in some samples was degraded as a follow-up RT-qPCR could detect COVID-19 in only 30.7% of the same sample set.

Nearly all studies (n = 12 out of 15) using RT-LAMP on crude patient samples reported less than 90% diagnostic sensitivity. The studies by Thi et al. (2020) and Lamb et al. (2020) reported even less than 50% sensitivity^39,57^. Such low sensitivity measure could be explained by the fact that these studies used patient samples with low viral load. Excluding positive samples with Ct = 30 or above, the calculated sensitivities from these studies rise above 60% (Table S4). Diagnosis of crude patient samples using non RT-LAMP assays has sensitivity ranging from 74 to 100%, with two exceptions. Basu et al. (2020)^29^ and Schermer et al. (2020)^75^ reported 55% and 56% diagnostic sensitivity for ID Now and CRISPR diagnosis, respectively. For the study by Schermer et al., all positive samples had Ct value below 30. This implies that poor sensitivity measure resulted from the performance of the assay itself and not because the samples had low viral load. Basu et al. did not show Ct value of samples used in their study^29^. Thus, it could still be possible that poor performance partially resulted from positive samples with low viral load. In fact, another study by Smithgall et al. showed that ID Now diagnostic sensitivity is 100% for samples with Ct value not exceeding 30 but only at 34.4% for samples with Ct value above 30^77^ (Table S4).

### Publication bias

Publication bias of all 81 included studies was determined using Deek’s funnel plot test for DOR. The result indicates significant asymmetry in funnel plot (p-value = 3.203 × 10^–4^).

### Meta-analysis of sensitivity, specificity and DOR

We performed subgroup analysis of all studies that used RT-qPCR as reference test and had at least ten positive and ten negative patient samples. The two outlier studies by Rohaim et al.^74^ and Li et al.^59^ were excluded from the subgroup analysis. If multiple studies were conducted on the same set of patient samples, only a study with the highest sensitivity and specificity was used. For example, Patchsung et al.^67^ reported two CRISPR diagnosis studies on the same set of patient samples, one using fluorescent readout and the other using lateral flow assay. In our analysis, we included only the fluorescent readout study, which had higher sensitivity and similar specificity to the lateral flow assay.

**Figure 4:**
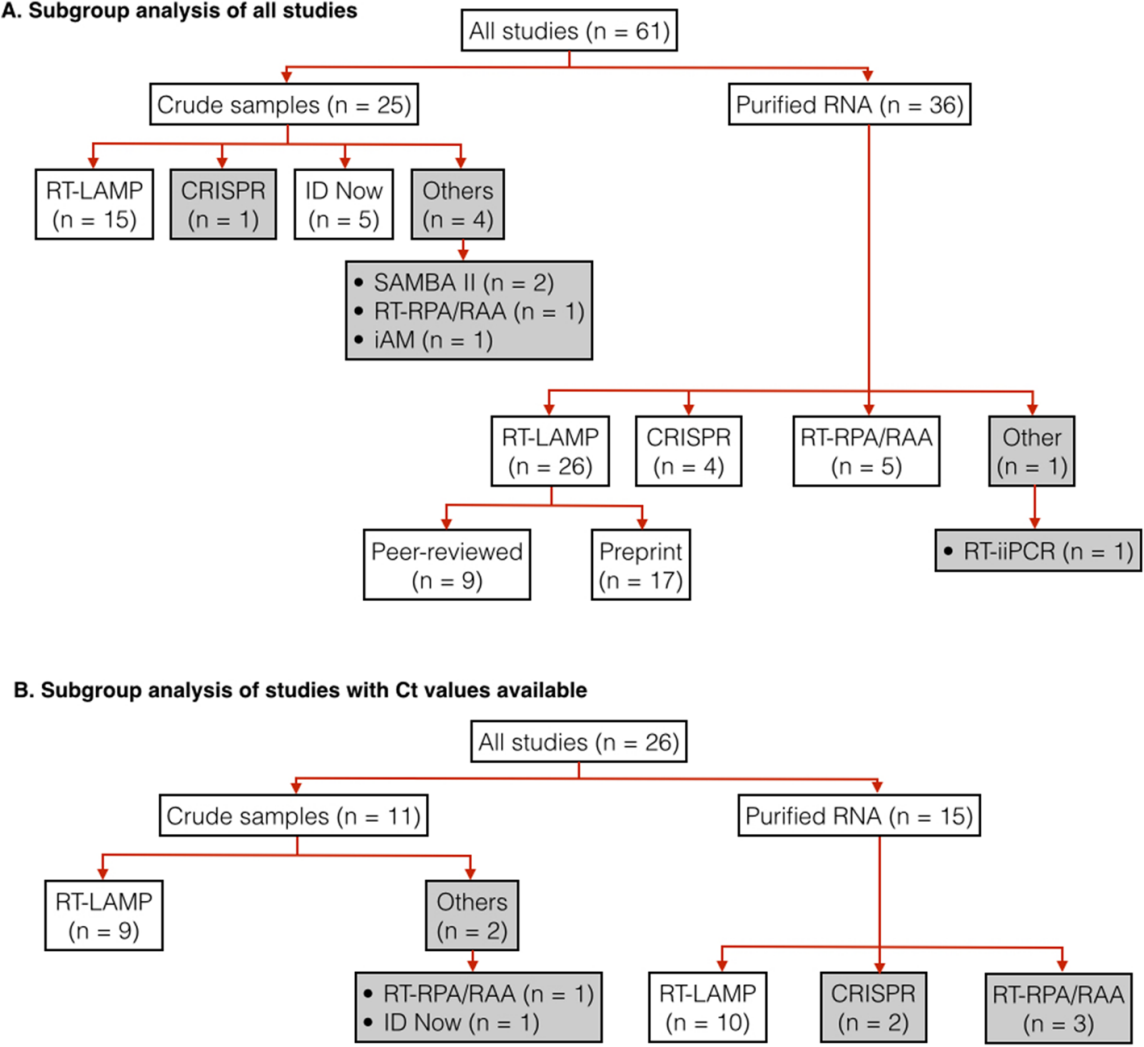
Hierarchical subgrouping of studies for meta-analysis. **(A)** subgrouping of all qualified studies. **(B)** subgrouping of only qualified studies that provide Ct values of positive samples. “n” indicates the number of studies in a subgroup. Pooled diagnosis results from subgroups in white boxes were used for calculating pooled sensitivity, specificity and DOR. Subgroups in grey boxes were not used for sensitivity, specificity, nor DOR calculation.

In total, 61 studies were used for subgroup analyses (Fig. 4A, Table S5). These studies were divided up further according to the types of samples used (purified RNA vs crude patient samples) and index test assays. We only estimated pooled sensitivity, specificity and diagnosis odds ratio for subgroups that had at least four studies. For the studies subgroup using RT-LAMP on purified RNA samples, we also performed a further subgroup analysis to compare the performance of studies from peer-reviewed and from pre-print articles.

Pooled sensitivity and specificity of all included studies are at 91% and 99%, respectively, indicating overall good performance of isothermal amplification based diagnosis test so far (Table 1). Pooled sensitivity of studies using purified RNA sample at 0.94 (95% CI: 0.92–0.96) is clearly higher than those using crude patient samples at 0.83 (95% CI: 0.74–0.89).

**Table 1:** Summary of sensitivity, specificity and DOR.

Subgroup (n)	Sensitivity	Specificity	ln(DOR)
**All studies (n = 62)**	0.91 (0.88–0.94)	0.99 (0.99–1.00)	7.29 (6.53–8.05)
Crude sample (n = 25)	**0.83 (0.74–0.89)**	0.99 (0.97–0.99)	5.78 (3.32–4.75)
RT-LAMP (n = 15)	*0.78 (0.65–0.87)*	0.96 (0.95–0.99)	4.96 (3.99–5.92)
ID Now (n = 5)	*0.74 (0.67–0.80)*	0.99 (0.97–1.00)	6.10 (4.58–7.62)
Purified RNA (n = 37)	***0.95 (0.92–0.96)***	1.00 (0.99–1.00)	8.33 (7.22–9.44)
RT-LAMP (n = 26)	0.94 (0.90–0.96)	1.00 (0.99–1.00)	8.49 (7.06–9.92)
Peer review (n = 9)	0.96 (0.88–0.99)	0.99 (0.98–1.00)	7.81 (6.46–9.16)
Pre-print (n = 17)	0.93 (0.89–0.96)	1.00 (0.98–1.00)	8.87 (6.41–11.32)
CRISPR (n = 4)	0.94 (0.89–0.97)	0.98 (0.91–1.00)	6.88 (4.75–9.02)
RT-RPA/RAA (n = 5)	***0.96 (0.93–0.98)***	1.00 (0.85–1.00)	8.99 (5.07–12.92)
**All studies with Ct (n = 26)**	**0.87 (0.80–0.94)**	0.99 (0.98–1.00)	6.96 (5.79–8.13)
Crude sample (n = 11)	*0.76 (0.57–0.88)*	0.98 (0.94–0.99)	4.95 (3.73–6.17)
RT-LAMP (n = 9)	*0.73 (0.51–0.88)*	0.97 (0.93–0.99)	4.57 (3.40–5.75)
Purified RNA (n = 15)	0.93 (0.87–0.97)	1.00 (0.99–1.00)	8.23 (6.78–9.67)
RT-LAMP (n = 10)	0.92 (0.82–0.97)	1.00 (0.99–1.00)	8.00 (6.80–9.20)
**All studies with Ct, excluding high Ct samples (n = 26)**	***0.99 (0.96–1.00)***	0.99 (0.98–1.00)	9.95 (7.78–12.13)
Crude sample (n = 11)	0.95 (0.84–0.99)	0.98 (0.94–0.99)	6.98 (5.15–8.81)
RT-LAMP (n = 9)	0.91 (0.79–0.97)	0.98 (0.94–0.99)	6.06 (4.65–7.48)
Purified RNA (n = 15)	***1.00 (0.96–1.00)***	1.00 (0.99–1.00)	12.09 (8.38–15.79)
RT-LAMP (n = 10)	1.00 (0.89–1.00)	1.00 (0.99–1.00)	12.04 (7.57–16.50)

The numbers in each cell show estimated pooled sensitivity, specificity and ln(DOR). The numbers in parentheses are 95% confidence intervals. For the sensitivity column, italics cells indicate expected pooled sensitivity values at 70–80%; bold, expected pooled sensitivity values at 80–90%; bold-italic, lower band for 95% confidence interval of pooled sensitivity values above 90%.

Similarly, pooled sensitivity of studies using RT-LAMP on purified RNA sample is clearly higher than that for studies using RT-LAMP on crude patient samples. Pooled sensitivity of RT-LAMP on crude samples was similar to that of ID Now. Both pooled sensitivities were lower than that of SAMBA II (Fig. 3, not used in subgroup analysis). For diagnosis of purified RNA samples, pooled sensitivities of RT-LAMP, CRISPR diagnosis and RT-RPA/RAA were similar.

Additionally, pooled sensitivity of RT-LAMP studies in peer-reviewed articles was not significantly different from that in pre-print articles.

The distribution of viral load in tested samples is one of the key factors that determine measured sensitivity of an index test. If a large fraction of positive samples used in a study have low viral load (i.e., high Ct value), measured sensitivity will be low. Unfortunately, the majority of our included studies (n = 35 out of 61) do not show Ct values of positive samples. Thus, it was not possible to determine the extent to which viral load in positive samples from these studies affected their measured sensitivity. For this reason, we decide to focus our analysis on only studies that reported Ct values.

In total, 26 studies were used for subgroup analyses (Fig. 4B, Table S5). Again, these studies were divided up further according to types of samples used (purified RNA vs crude patient samples) and index test assays. For each subgroup analysis, we calculated pooled sensitivity, specificity and DOR for the cases when all samples were used and the cases when positive samples with high Ct values were excluded. The ‘high’ Ct cut-off values were not the same in all included studies (depending on data available from the original research articles). Most studies (n = 19 out of 26) had Ct cut-off values of 30–33; the remainder had Ct cut-off values of 34–39 (Table S4). Excluding samples with high Cts from the calculation resulted in substantial increases in sensitivity, particularly for diagnosis of crude samples (Table 1, Table S4). The calculated pooled sensitivity for crude samples increases from 0.76 (95% CI: 0.57–0.88) to 0.95 (95% CI: 0.84–0.99); the calculated pooled sensitivity for RT-LAMP on crude samples increases from 0.73 (95% CI: 0.51–0.88) to 0.91 (95% CI: 0.79–0.97). Diagnostic sensitivity of purified RNA samples remained higher than those of crude patient samples. However, when positive samples with high Ct values were excluded, such difference in sensitivity become smaller.

## Discussion

To our knowledge, this is the first systematic review and meta-analysis examining the performance of isothermal nucleic acid POCTs for human coronavirus. The majority of studies that used purified RNA for diagnosis reported at least 90% sensitivity and specificity; over a third of these studies reported 100% sensitivity and specificity. Sensitivities were generally lower for studies that used crude patient samples for diagnosis while specificities were not substantially different. Subgroup analyses confirmed the difference in sensitivity between diagnostic test performed on purified RNA and on crude patient samples. Nonetheless, when positive samples with low viral loads (Ct value = 30 or above) were excluded from calculation, such difference become much smaller. In other words, for samples with medium to high viral load, coronaviruses could be reliably detected directly from crude patient samples without an RNA purification step which takes more time and technical expertise.

Almost two thirds of the included studies used RT-LAMP as an index test. At the time of this writing, the only published meta-analysis of RT-LAMP performance was on a diagnostic accuracy of Enterovirus 71 by Lei et al. (2014)^89^. That meta-analysis included 907 clinical samples from ten studies, all performed on purified RNA. Pooled data had a sensitivity of 0.99 (95% CI: 0.97–1.00), specificity of 0.97 (95% CI: 0.94–1.00) and ln(DOR) of 6.74 (95% CI: 5.68–7.79). Our subgroup analysis showed that RT-LAMP on purified RNA samples had pooled sensitivity of 0.94 (95% CI: 0.90–0.96), specificity of 1.00 (95% CI: 0.99–1.00) and ln(DOR) of 8.49 (95% CI: 7.06–9.92) (Table 1). Pooled sensitivity of RT-LAMP from our analysis appeared to be lower. Nonetheless, when samples with high Ct values were excluded, pooled sensitivity of analyses went up to 1.00 (95% CI: 0.89–1.00) which is within the same range as the analysis by Lei et al. Thus, our reported RT-LAMP performance is likely to reflect the true performance of this isothermal nucleic acid test as the performance value is generalizable across different target viruses. This could serve as a reference point for assessing the performance of other diagnostic methods.

Among RT-LAMP studies on purified RNA, the studies by Hu et al., Kitakawa et al., Thi et al. and Yan et al.^39,49,55,84^ were of high quality: they tested large sample sizes and had no QUADAS-2 domain with high risk of bias or concern of applicability (Table S2). Yet, these four studies reported contrasting results with respect to diagnostic performance. While Kitakawa et al.^55^ and Yan et al.^84^ demonstrated 100% diagnostic sensitivity, Thi et al.^39^ reported only 70% sensitivity. Generally, the measured false negative rate of a diagnosis test is high when the viral loads in the majority of tested samples are low.

For example, Thi et al. showed that RT-LAMP sensitivity is at 100% when the samples have viral RNA concentration equivalent to Ct of 0–25 cycles. The sensitivity decreases to about 30% at RNA concentration equivalent to Ct of 30–35 cycles and to sensitivity less than 6% at RNA concentration equivalent to Ct of 35–40 cycles. Approximately a third of positive samples in Thi et al. study has Ct of 30–40 cycles. This could explain why RT-LAMP in this study appear to have such a high false negative rate. Yan et al. and Kitakawa et al. did not report the distribution of viral RNA level in their tested samples. Thus, it is possible these two studies appear to achieve 100% sensitivity simply because most of their positive samples had high viral RNA level.

Viral RNA levels in samples depend on several factors including severity of the disease, timing of sample collection, types of samples and sample handling processes. Without such information, it is difficult to determine whether the difference in observed sensitivity results from the performance of the test itself or properties of the samples used in the test. Unfortunately, most included studies provided no information about viral RNA levels in the infected samples (as determined by a standard reference test, e.g., RT-qPCR). Information about disease severity and sample collection timing (i.e., days after disease onset) are often missing. Future works should provide this information in order to allow a better assessment of diagnosis test performance and must identify their actual limitations.

Of all included studies in this review, only two studies by Schermer et al. (2020) attempted to directly compare the coronavirus detection accuracy of CRISPR diagnosis to that of RT-LAMP using the same set of clinical samples^75^. They estimated diagnostic sensitivity (in crude patient samples) of RT-LAMP at 73% and of CRISPR diagnosis at 56%. While the sensitivity of RT-LAMP shown in this study was on par with RT-LAMP performance reported by other studies in our review, diagnosis sensitivity by CRISPR was surprisingly low. Other studies including Arizti-Sanz et al. (2020) and Joung et al. (2020) estimated sensitivity of CRISPR diagnosis on crude samples at 90–100%^26,52^. From Schermer et al. study, CRISPR diagnosis failed to detect any positive samples with Ct values above 21 cycles. In contrast, the study by Joung et al. (2020) using CRISPR diagnosis could reliably detect all positive samples with Ct of 20–35 cycles^52^. For coronavirus detection in purified RNA samples, estimated sensitivity of RT-LAMP and of CRISPR diagnosis were almost identical according to our subgroup analysis (Table 1). Given available data, the difference in performance of CRISPR diagnosis and RT-LAMP remains inconclusive. Existing CRISPR diagnosis also required RT-LAMP or other isothermal techniques to pre-amplify nucleic acid targets before CRISPR detection. The use of cas12 or cas13 enzyme adds to the cost of CRISPR diagnosis test kit, making it likely to be more expensive than RT-LAMP. Future studies should directly compare and highlight the unique strength of CRISPR diagnosis relative to other isothermal techniques, for example, its ability for multiplex detection and identifying single base differences in targeted genomes^90–92^.

All seven studies using RT-RPA/RAA as an index test reported over 90% sensitivity and at least 98% specificity. Additionally, the studies by Qian et al. (2020) showed that RT-RPA/RAA based diagnosis on crude patient samples could achieve detection sensitivity level similar to diagnosis on purified RNA samples^70^. While most studies did not report Ct value of positive samples, one study by Wu et al. (2020) demonstrated that RT-RPA/RAA based diagnosis can detected 91% (30 out of 33) positive samples with Ct values of 30–36 cycles^82^. Together, the results from these studies suggested that RT-RPA/RAA could potentially be one of the most promising approaches for developing coronavirus POCTs. Future works should directly compare this assay to other nucleic acid POCTs such as RT-LAMP using the same sample sets in order to determine the actual difference in their diagnosis performance.

Besides RT-LAMP, CRISPR diagnosis and RT-RPA/RAA, two other diagnostic assays with over a hundred positive and negative pooled samples in our review were Abbott ID Now and SAMBA II. Abbott ID Now is famous for being “the fastest” (5–13 min) isothermal COVID-19 nucleic acid detection system in the market. However, four out of five ID Now studies included in our review reported less than 80% sensitivity. Pooled data from ID Now studies have sensitivity levels on par with that of RT-LAMP applied to crude samples. Only one of these five studies by Smithgall et al. (2020) showed Ct values of positive samples^77^. The study reported 74% sensitivity when all samples were used for calculation. For samples with Ct below 30 cycles, the sensitivity is 100%. Thus, the poor performance of ID Now reported in these studies could partly result from having the large proportion of positive samples with low viral loads. The two SAMBA II studies each included over a hundred tested samples and had no high risk or concern QUADAS-2 domains^27,38^. In these two studies, sensitivity and specificity at 97% or above were among those with highest accuracy of all the included studies in our current systematic review. Despite being the slowest POCTs among our included studies (> 1 h from sample to readout), SAMBA II is arguably one of the most promising POCTs thus far regarding diagnostic accuracy for coronavirus detection of crude patient samples. Unfortunately, neither of these two studies reported Ct values of the samples used. Thus, given data availability for this review, we still cannot directly compare the performance of this approach to other assays.

Our study identified both relevant peer-reviewed studies and preprints for deriving better scientific conclusions in diagnosis of the life-threatening novel coronavirus in a timely manner. Our study also adhered to the standard methodology of systematic review and meta-analysis as indicated by the PRISMA-DTA statement^93^. However, our study had few limitations. First, almost half of the included studies (n = 38 out for 81) had high risk of patient selection bias or index test bias. Such bias could lead to over-estimation of diagnosis performance. Nonetheless, the studies that reported the highest performance (near 100% sensitivity and 100% specificity with narrow 95% CIs) were also the ones with lowest QUADAS risk and concerns in all domains^27,38,41,45,55,82–84^. Second, almost two thirds (n = 28) of included studies had not been peer-reviewed. Nevertheless, our analysis showed that sensitivity and specificity for pooled data from these preprint manuscripts was not significantly different from that of the peer-reviewed studies. Therefore, an inclusion of data from preprint manuscripts is unlikely to skew the results of our other analyses. Given that the peer-review process often takes at least a few months, the systematic review that includes preprint manuscripts is timely for guiding the direction of on-going research especially during a global pandemic. Third, our analysis indicated significant publication bias in included studies. Given that all nucleic acid isothermal POCTs for coronaviruses were still in an early stage of development, nearly all reported diagnosis accuracy assessments were performed and published only by the same research groups that developed or optimised the assays. Thus, there is likely to be bias toward reporting higher diagnosis performance. We expected that such bias would be mitigated once these POCTs are fully deployed in the field and assessed by multiple and independent research teams, not directly affiliated with POCT developers.

We identified a recent comprehensive systematic review on nucleic acid POCTs of SARS-CoV-2 by Dinnes et al. (2020)^94^. The review by Dinnes et al. was published after our preprint version was available online^95^. Dinnes et al. searched article database only up to 25 May 2020 and did not include any isothermal POCT assays besides ID Now. While the systematic review by Dinnes et al. was more selective for studies that were highly relevant to ongoing clinical uses, our review offered a broader perspective on the performance of diverse competing diagnostic platforms.

In conclusion, our systematic review and meta-analysis revealed the current state of nucleic acid POCTs for human coronaviruses. Overall diagnostic accuracy of these POCTs reported so far was high but the quality of these studies was still in question. Particularly, future study should attempt to use un-bias (e.g., random or consecutive) patient cohorts and perform double-blinded index test. Such improvement in study design and methodology would enhance validity of the estimated sensitivity and specificity of POCTs. This would allow researchers and healthcare providers to make correct decisions on which POCTs platforms to deploy or upgrade.

Sensitivity and specificity of RT-LAMP, RT-RPA/RAA and CRISPR diagnosis on purified RNA samples were not materially different. Critical information about viral load or factors influencing viral load was missing in most studies. It is still unclear whether CRISPR diagnosis was superior to a cheaper, simpler and more established nucleic acid POCTs such as RT-LAMP. The performance of viral detection directly from patient samples is substantially lower than from purified RNA.SAMBA II had highest diagnostic accuracy among all POCTs for crude samples in this systematic review while Abbott ID Now had lower diagnostic accuracy. A breakthrough in bypassing an RNA purification step will simplify the workflow, reduce time, cost and possible errors. The improvement in these key areas will bring nucleic acid POCTs toward large practical uses for surveillance of on-going and future coronavirus outbreaks.

## Supplementary Information

Supplementary Tables.
